# Managing cataract surgery in patients with small pupils

**Published:** 2019-02-10

**Authors:** Mariano Yee Melgar, John Buchan

**Affiliations:** 1 Ophthalmologist: Visualiza, Guatemala, Central America.; 2 Ophthalmologist: International Centre for Eye Health, London School of Hygiene and Tropical Medicine, London, UK.

**Cataract surgery is more difficult when a patient has a small pupil, but optimising pharmacological dilation and adapting your surgical technique can ensure good outcomes.**

**Figure:**
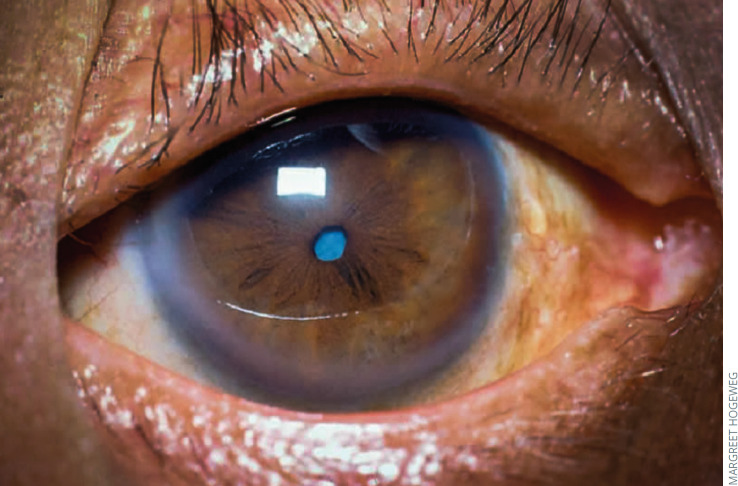
Small pupil.

Removing a cataract, typically around 10 mm in diameter, is made much more difficult when a patient has a small pupil; the risk of rupture of the posterior capsule during surgery is also 50% higher.^1^ Pre-operative action can sometimes improve this situation, but the solution frequently involves changes in surgical technique.

## Before surgery

Take a careful history and ask patients about the medication they are currently using.

- **Oral alpha blocker medication**, such as tamsulosin or doxazosin, help urinary symptoms by relaxing the smooth muscles of the bladder neck. They also affect the iris, causing poor dilation and intraoperative floppy iris syndrome (IFIS). The amount of IFIS does not seem to be related to the dose or the duration of the therapy,^3^ so stopping these medications, even several months before surgery, often does not reverse this effect.
- Patients using **topical pilocarpine** should be asked to stop at least three weeks before surgery.

**Pre-operative NSAID drops**, such as ketorolac, nepafenac or diclofenac, instilled 30–90 minutes before surgery, have been shown to maintain pupil dilation during surgery.

Where **posterior synechiae** are present, consider whether these were caused by uveitis, rather than by previous infection, surgery or trauma. If they were caused by uveitis, aggressive management of peri-operative and postoperative inflammation will be essential for obtaining a good outcome. Careful consideration should be given to IOL choice, or even aphakia, especially in younger patients with uveitis (pp. 82–83).

## Pharmacological dilation

**Avoid dilating more than 1–2 hours before surgery** as the dilation effect wears off and subsequent drops work less well.

**Optimal dilation** can be promoted by putting little pieces of surgical sponge or cotton wool soaked in 10% phenylephrine, or a mixture of phenylephrine and cycloplegic eyedrops, into the inferior fornix 30 minutes before surgery. Be sure to remove these before the procedure.

### Maintaining dilation

Many eye units routinely add 0.5 ml of preservative-free adrenaline (1 mg/ml) to 500 ml of balanced salt solution or Ringer's lactate to help maintain dilation during cataract surgery; for patients who have a small pupil or a floppy iris, stronger intracameral agents can be used.

Phenylephrine (prepared from 2.5% or 10% preservative-free drops) can be prepared with concentrations ranging from 0.5% to 1.5%. Adequate mixing of agents must be ensured by using 2.5 ml or 5 ml syringes only (avoid 1 ml syringes). It may be preferable to use 10% phenylephrine as this ensures greater dilution of other agents contained in the drops.

Example preparation guidelines might be:

- 2 drops (approximately 0.1 ml) of 10% preservative-free phenylephrine added to 1–2 ml of balanced salt solution, or
- 0.5 ml of 2.5% preservative-free phenylephrine diluted with 1 ml balanced salt solution.

If additional anaesthetic is also desired, a version of epi-Shugarcaine^2^ can be produced as follows:

- 9 ml of balanced salt solution
- 3 ml of 4% preservative-free lidocaine
- 4 ml of 1:1,000 preservative-free, bisulfite-free epinephrine.

If you need a smaller quantity, mix 1 ml of 4% preservative-free lidocaine with 3 ml of balanced salt solution, then discard 1 ml of this mixture and add 1 ml of preservative-free epinephrine.

## Surgical/mechanical dilation

Even after pharmacological interventions, many patients will still have pupils that are too small.

If the pupil is fixed with posterior synechiae, you will need to break them with an iris spatula or with any other blunt instrument.

If the pupil size is fixed due to fibrosis, try to remove the fibrotic tissue using Utrata forceps.

If those measures are not sufficient, then you will have to enlarge the pupil mechanically. There are several options for doing this.

### 1 Pupil stretch

Stretch the pupillary sphincter using two instruments (such as Lester hooks) through two anterior chamber paracenteses. Engage the pupillary margin at opposite points and stretch the pupil to the limbus for a few seconds. This can be repeated 90° apart. Some bleeding can result, and the pupil may be permanently dilated or distorted.

### 2 Radial iridotomy

To preserve a round pupil, a radial iridotomy can be made. First, make a small peripheral iridectomy and then extend the cut to the pupillary margin. Suture afterwards using 10-0 non-absorbable interrupted sutures (Figure 2). The procedure demands considerable skill and patience.

**Figure 1:**
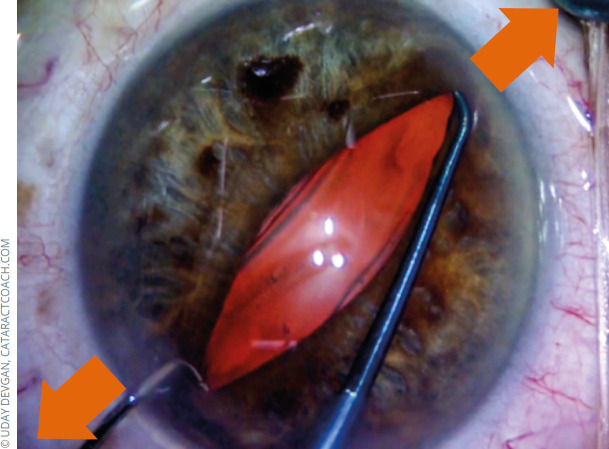
Place the instruments 180°apart and stretch the pupil towards the limbus, as shown^4^

**Figure 2:**
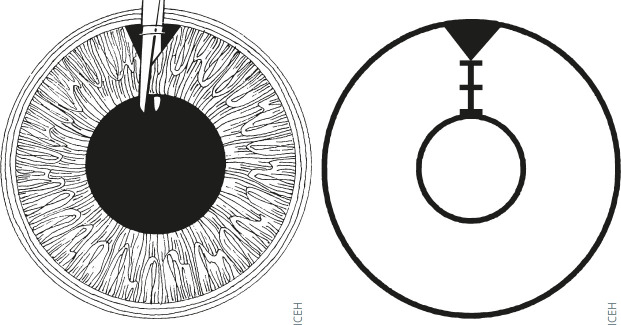
Radial iridotomy (left). Suture with 10.0 interrupted sutures (right)

### 3 Sphincterotomy

**Figure 3:**
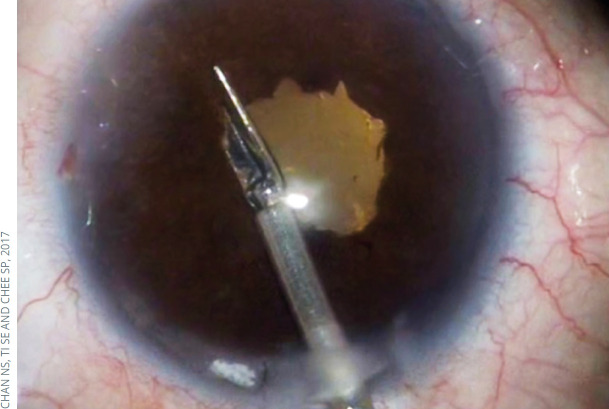
Sphincterotomy

Make several small cuts on the pupillary sphincter to allow room for the cataract to pass through (Figure 3). After the small cuts are made, deepen the anterior chamber with viscoelastic to achieve the dilation. A sphincterotomy is usually not necessary if you stretch the pupil.

### 4 Iris hooks

Use four or five iris hooks, spaced evenly apart. You can make your own ones using 27- or 30-gauge cannulae and pieces of rubber. However, it is complicated to make them yourself, and potentially expensive to purchase, so pupil stretching is often preferable.

### 5 Other devices

Other devices, such as Malyugin rings, can enlarge the pupil to 6.25 or 7 mm, which is large enough for phacoemulsification but often not for SICS or ECCE.

## Postoperative care

When any surgical intervention is used to mechanically dilate the pupil, expect an increase in postoperative inflammation and take post-operative anti-inflammatory measures, such as sub-conjunctival steroid injection at the end of the procedure, using a more potent topical steroid postoperatively, or instilling drops more frequently (e.g., dexamethasone 0.1% or prednisolone 1% six times a day).

**Figure 4:**
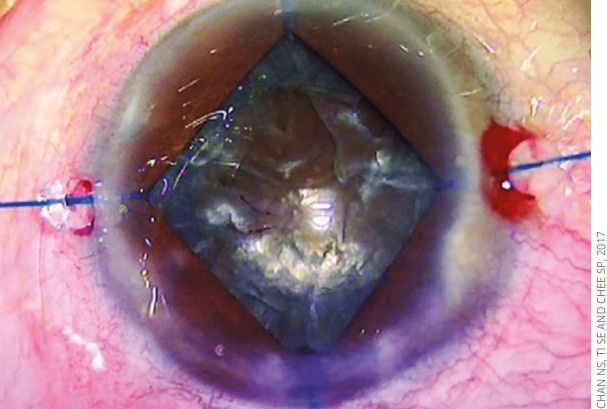
Iris hooks
